# The Diagnostic Efficacy of Flexible Fiberoptic Laryngoscopy and Its Correlation With Histopathology in Different Benign Lesions of the Vocal Cord in a Tertiary Care Hospital: A Prospective Clinical Study

**DOI:** 10.7759/cureus.76230

**Published:** 2024-12-22

**Authors:** Agalya P Vasanthi.L, Anbukarasi Ramalingam, Nithya Narayanan

**Affiliations:** 1 Otorhinolaryngology-Head and Neck Surgery, Apollo Hospitals, Chennai, IND; 2 Otorhinolaryngology-Head and Neck Surgery, Panimalar Medical College Hospital and Research Institute, Chennai, IND

**Keywords:** benign vocal cord lesions, flexible fiberoptic laryngoscopy, histopathology, vocal nodule, vocal polyp

## Abstract

Introduction

Benign vocal cord lesions are diagnosed by clinical examination with usually an office-based laryngoscopy examination. The severity of voice impairment can be assessed by severity scores such as the Voice Handicap Index (VHI). These lesions are usually treated by conservative methods such as voice rest/restriction and voice therapy. A clinically benign-looking lesion does not always exclude the presence of malignancy. A mere clinical diagnosis of a benign vocal cord lesion is not complete, and hence, a confirmed diagnosis is made only after excision and histopathological examination (HPE) of the lesion.

Aim

This study was primarily aimed at knowing the prevalence, etiological factors, and clinical presentation of patients with benign vocal cord lesions, assessing the diagnostic potential of flexible fiberoptic laryngoscopy (FFL) in different benign lesions of the vocal cord and its correlation with histopathology, and studying the symptomatology and severity of symptoms in different benign vocal cord lesions using the Vocal Handicap Index-10.

Method

A prospective study of benign lesions of the vocal cord was conducted from January 2018 to June 2019 on 125 cases in a tertiary care hospital. All the benign lesions were clinically diagnosed with flexible fiberoptic laryngoscopy, and their findings were correlated with histopathology examination.

Results

Of the 125 patients, the overall prevalence of benign vocal cord lesions was higher in male patients (n = 88 (70.4%)) than in female patients (n = 37 (29.6%)). Voice abuse is the major risk factor for benign vocal cord lesions. The most common lesion identified by flexible fiberoptic laryngoscopic (FFL) examination is polyps (61%), followed by nodules (14%) and keratosis (13%). On the correlation of clinical diagnosis with histopathological examination, the diagnostic accuracy of FFL is 51.8% for nodules, 44.4% for polyps, 35.5% for keratosis, and 75% for granulomas. The diagnostic accuracy of FFL examination in diagnosing truly benign lesions is 94.9%.

Conclusion

Benign vocal cord lesions are one of the common cases seen in ENT practice. Voice abuse was the predominant causative factor for all these lesions. In our study, vocal polyp was the most common lesion by clinical examination using FFL. On correlation of fiberoptic laryngoscopic diagnosis with histopathological examination, the diagnostic accuracy of FFL examination in diagnosing truly benign lesions is 94.9% in our study.

## Introduction

Voice is one of the most important components of one's identity. Voice is the most critical component of any communication system because it creates a personal connection between people. Voice problems can produce significant impairment of one's emotional, vocational, and social well-being. People with voice problems such as voice hoarseness, voice change, and voice fatigue immediately visit a physician, most commonly an otolaryngologist. In normal phonation, mechanical stress was least at the midpoint of the membranous vocal cord and highest at tendon attachments. In contrast, during hyperfunctional dysphonia, there was an increase in the vibration, resulting in the incomplete approximation of the vocal cords posteriorly and increased stress between the vibratory segments (the midpoint of the membranous vocal cords encounters higher shearing stresses) [1]. Many lesions can result from this process, including nodules, polyps, and cysts, but other pathology should be considered, such as reactive lesions, intracordal scarring, feeding varices, and reparative granuloma. The presence of the mass causes impaired vocal phase closure during phonation, resulting in excess air egress. Clinically, this not only adds to the breathy quality of the voice but also contributes to vocal fatigue. Disruption of vocal cord vibration and phase closure often leads to phase asymmetry (depending on the specific lesion), which adds to the grainy quality of the voice [1].

Some of the common vocal cord lesions are vocal nodules, vocal polyps, Reinke's edema, contact ulcer, intubation granuloma, vocal cord cysts, leukoplakia, papilloma, and keratosis [2]. These lesions are diagnosed clinically based on careful history-taking, clinical examination, and usually an office-based laryngoscopy examination. Various clinical diagnostic methods such as stroboscopy, videokymography, optical coherence tomography (OCT), CT scan, MRI, high-speed photography, contact endoscopy, and rigid telescope have been used for identifying vocal cord lesions [3]. We used flexible fiberoptic laryngoscopy (FFL), which provides the close visualization of vocal cords with local anesthesia and is most convenient and non-invasive.

The severity of voice impairment can be assessed by severity scores such as the Voice Handicap Index (VHI) or Dysphonia Severity Index (DSI) [4]. These lesions are treated usually by voice rest/restriction and voice therapy. These lesions are excised if it looks suspicious for malignancy. A clinically benign-looking lesion does not always exclude the presence of malignancy. A mere clinical diagnosis of a benign vocal cord lesion is not complete, and a confirmed diagnosis is obtained only after excision and histopathological examination (HPE) of the lesion. Hence, this study aimed to know the prevalence, etiological factors, and clinical presentation of patients with benign vocal cord lesions and assess the diagnostic potential of flexible fiberoptic laryngoscopy in different benign lesions of the vocal cord and its correlation with histopathology.

## Materials and methods

This prospective observational study was conducted in Apollo Main Hospitals, Chennai, India, from January 2018 to June 2019 on 125 cases, in the age group of 18-70 years. All patients diagnosed with benign lesions of vocal cords by flexible fiberoptic laryngoscopy and patients with symptoms such as hoarseness, voice fatigue, and foreign body sensation in the throat were included in the study. Old debilitating patients, patients with vocal cord palsy, all patients suspected to have malignant lesions of vocal cords by fiberoptic laryngoscopy, and all patients with voice problems due to infective causes and speech defects due to central nervous system lesions were excluded from the study.

Patients were first seen in ENT OPD at Apollo Main Hospitals, Chennai. Written and informed consent were obtained. Detailed history and ENT examination include an indirect laryngoscopy (IDL) examination. The larynx of the patient was examined using flexible fiberoptic laryngoscopy (FFL). The severity of symptoms was assessed using the Voice Handicap Index-10.

Procedure

In a sitting position, 10% lidocaine local anesthetic spray was sprayed on the patient's nose and pharynx. The patient was allowed to wait for 3-5 minutes. FFL was done trans-nasally. After examining the nasal cavity, nasopharynx, and oropharynx including the base of the tongue and vallecula, the larynx was examined. In the larynx, the areas visualized are the epiglottis, aryepiglottic folds, arytenoids, vocal cords, and subglottis. Vocal cords are examined during phonation and different phases of respiration. Bilateral pyriform sinus are examined. Any abnormality detected in the aforementioned areas is captured and documented. A clinical diagnosis is made at this stage, and the patients were advised conservative voice therapy or surgical excision accordingly. Those patients for whom surgical modality of treatment was planned underwent microlaryngoscopy and excision of the lesions under general anesthesia, and the specimen was sent for histopathological examination (HPE).

Data analysis

Data entry was done in an MS Excel spreadsheet (Microsoft Corp., Redmond, WA). Data analysis was carried out using the Statistical Package for the Social Sciences (SPSS) version 25.0 (IBM Corp., Armonk, NY). p values less than 0.05 are considered statistically significant.

Ethical approval

The study protocol received approval from the Institutional Human Ethics Committee-Clinical Studies of Apollo Hospitals, Chennai, under reference number ECR/37/Inst/TN/2013/RR-16.

## Results

**Figure 1:**
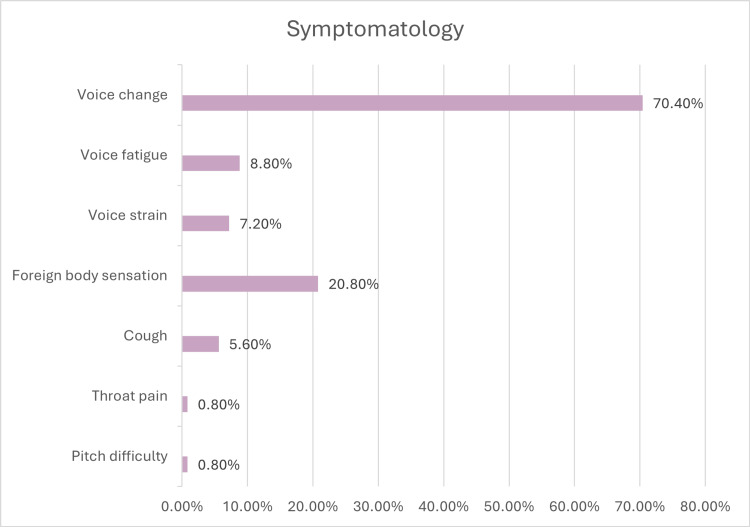
Bar diagram showing the percentage of patients with individual symptoms

Of the 125 patients, the overall prevalence of benign vocal cord lesions was higher in male patients (n = 88 (70.4%)) than in female patients (n = 37 (29.6%)). However, the prevalence of nodules and Reinke's edema was higher in female patients than in male patients. Among the 125 patients, four (3.2%) were less than 29 years, 35 (28%) were from 30 to 39 years, 50 (40%) were from 40 to 49 years, 24 (19.2%) were from 50 to 59 years, and 12 (9.6%) were from 60 to 70 years. Most of the patients with benign vocal cord lesions were business people (33.6%) who do sales work predominantly, followed by teachers (24%), and then housewives (16.8%). The symptomatology of benign vocal cord lesions is shown in Figure 1.

**Figure 2:**
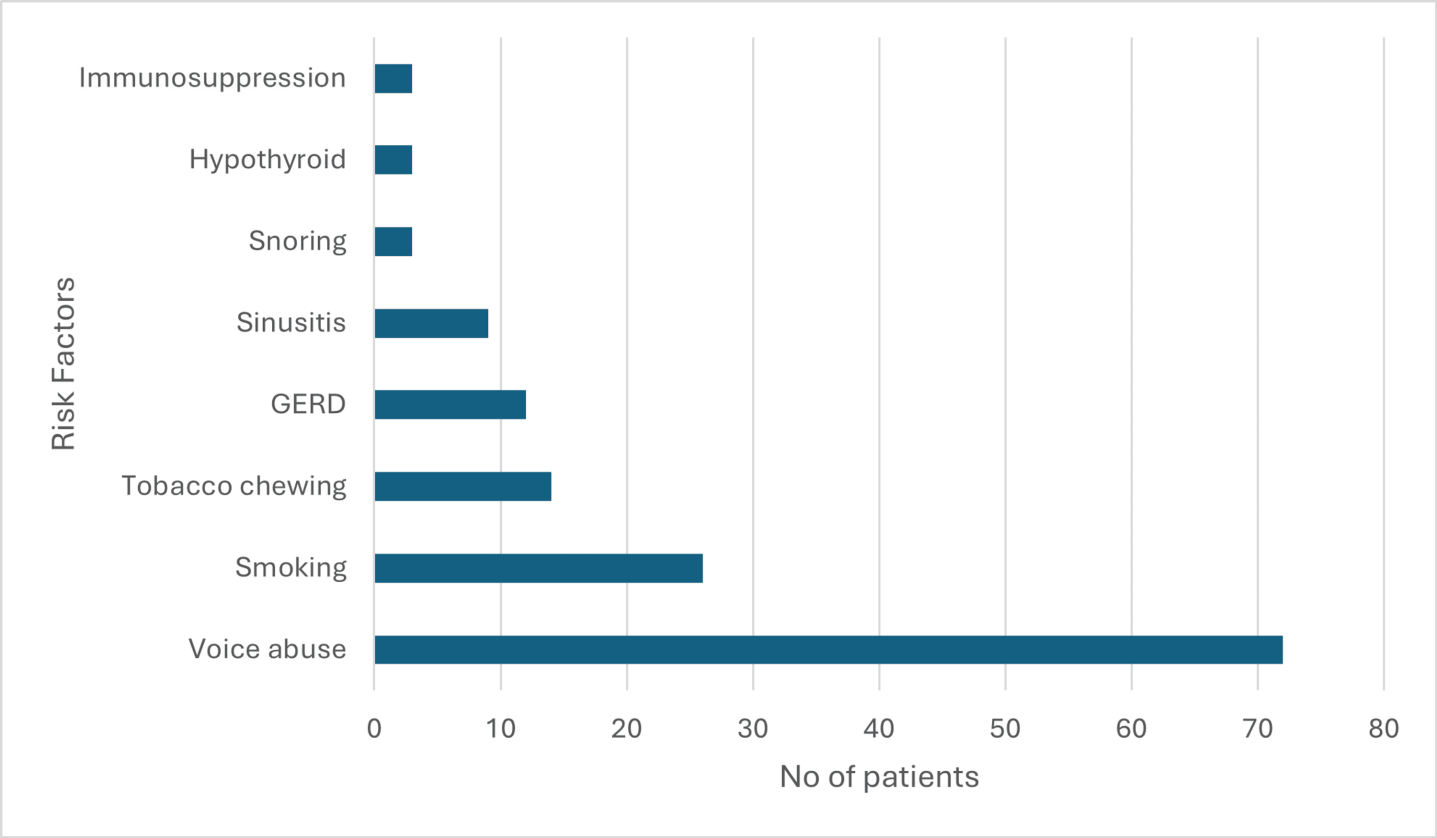
Bar diagram showing the risk factors of benign vocal cord lesions GERD: gastroesophageal reflux disease

The risk factors of benign vocal cord lesions in our study are shown in Figure 2.

**Figure 3:**
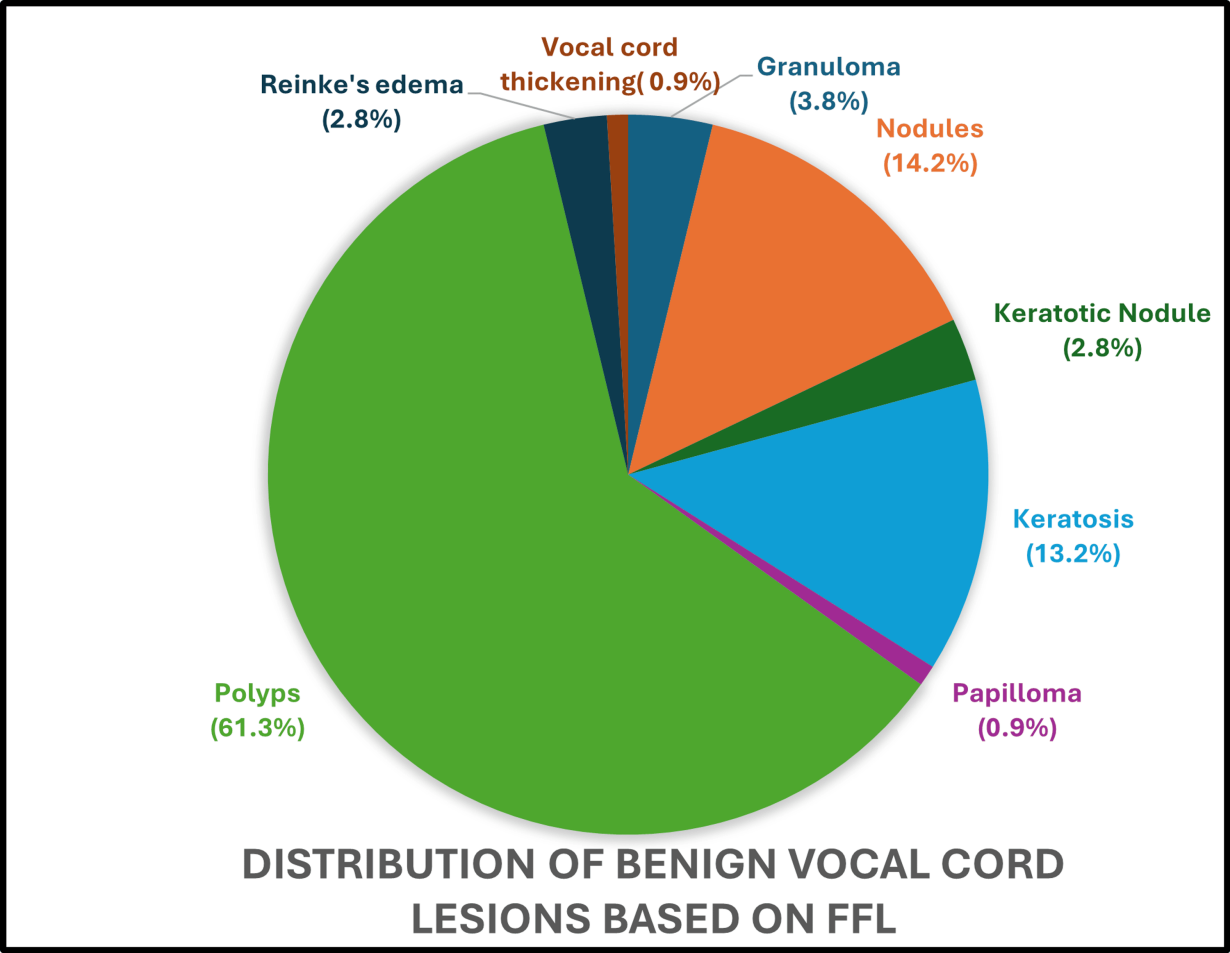
Distribution of benign vocal cord lesions based on FFL FFL: flexible fiberoptic laryngoscopy

Upon indirect laryngeal mirror (IDL) examination, the most frequent lesion identified was polyp (66%), followed by vocal nodules (15.2%), and keratosis (9.6%). VHI-10 score was abnormal in 122 (97.6%) out of 125 lesions. The mean VHI-10 score in patients with vocal cord nodules is 26.33%, while those with vocal cord polyps had a mean score of 23.36%. The most common lesion identified by FFL examination is polyps (61%), followed by nodules (14%), and keratosis (13%) (Figure 3).

**Figure 4:**
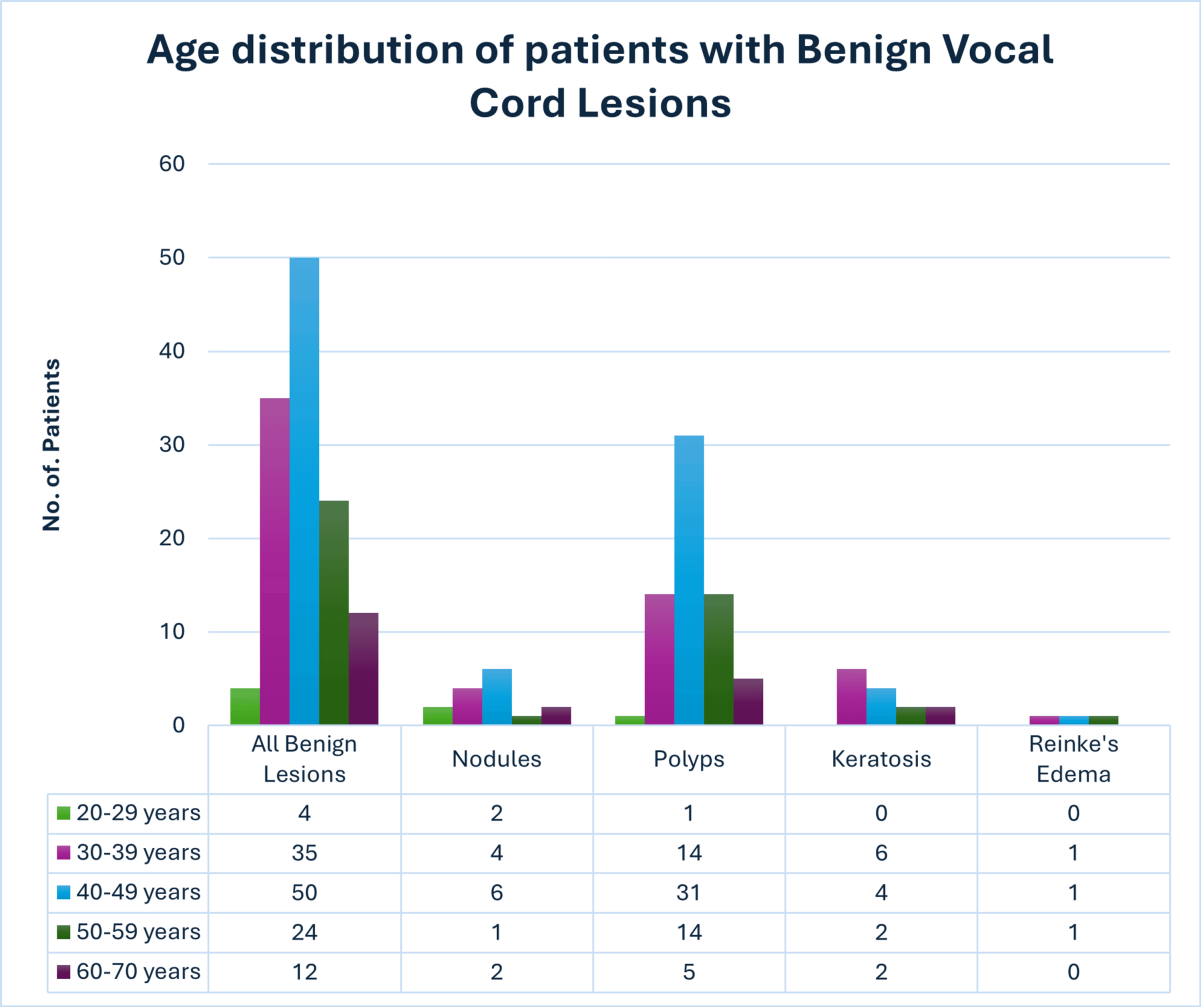
Age distribution of patients with benign vocal cord lesions

In our study, the overall prevalence of benign vocal cord lesions was higher in male patients than in female patients. However, the prevalence of nodules and Reinke's edema was higher in female patients than in male patients. The age distribution of patients with different benign lesions of the vocal cord is shown in Figure 4.

**Figure 5:**
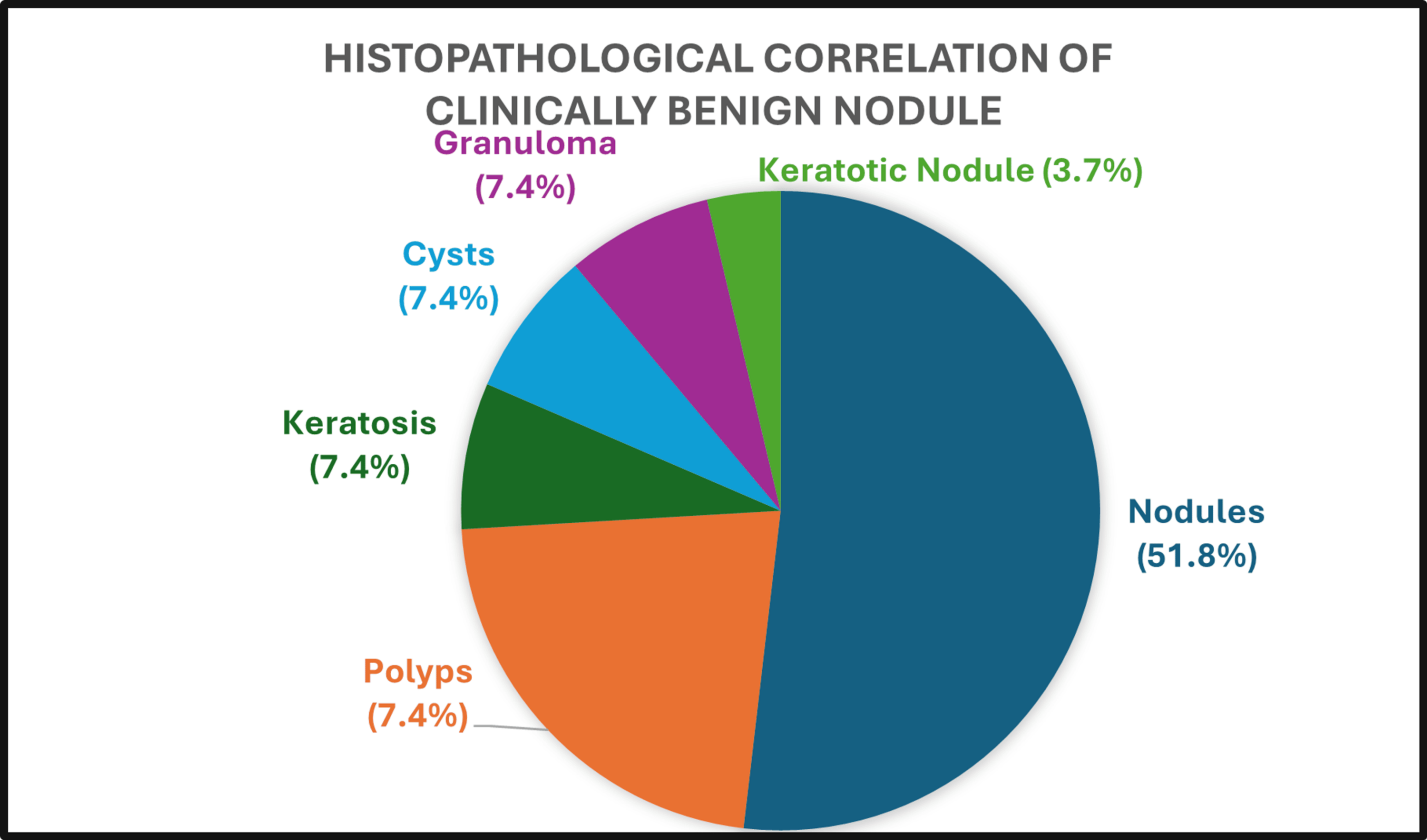
Histopathological correlation of clinically benign nodule

A total of 118 patients underwent surgery, and 159 lesions were excised during microlaryngoscopy and sent for histopathological examination. The remaining seven patients refused surgery and were treated with conservative voice therapy. Of the 159 lesions assessed, 27 lesions were clinically diagnosed as vocal nodules by FFL, and their HPE diagnoses were given in Figure 5. The accuracy of FFL examination in diagnosing a vocal cord nodule is 51.8%.

**Figure 6:**
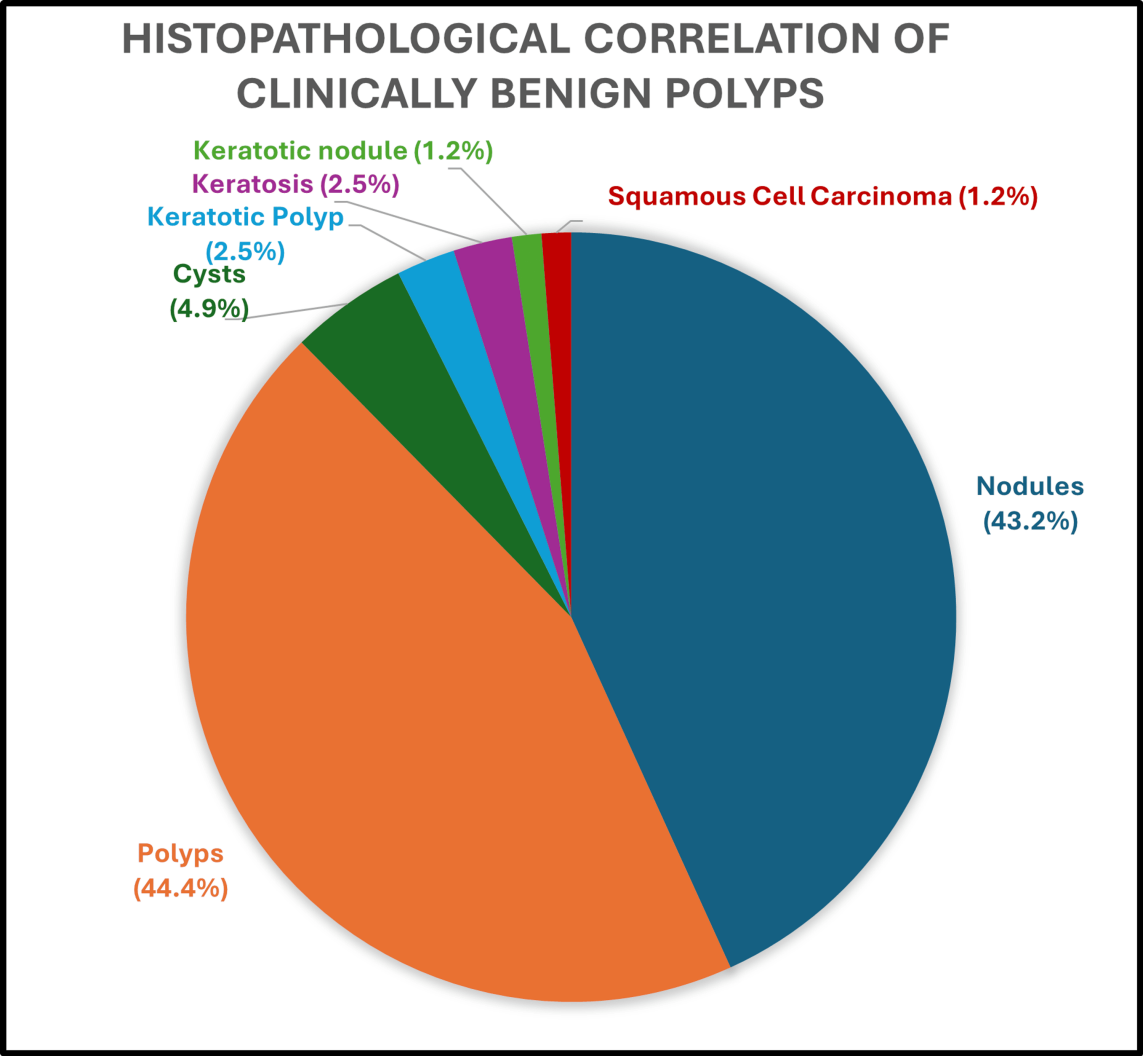
Clinical and histopathological correlation of benign vocal cord polyp

**Figure 7:**
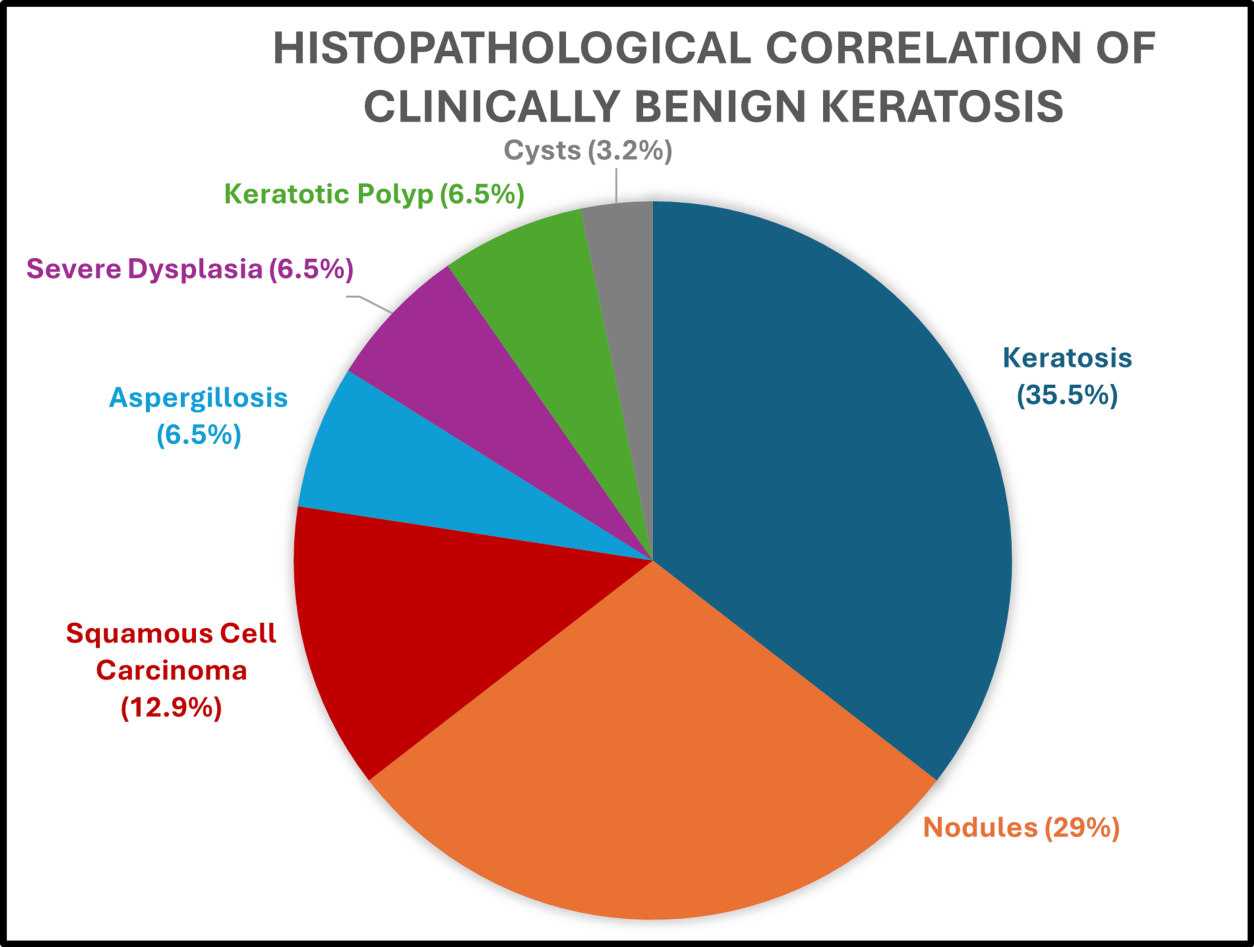
Histopathological correlation of clinically benign keratosis

Of the 159 assessed lesions, 81 were clinically diagnosed to have vocal cord polyps through fiberoptic laryngoscopy. Figure 6 shows the histopathological confirmation of the polyp and other lesions' distribution. The diagnostic accuracy of FFL in diagnosing vocal polyps is 44.4%. Of the 159 lesions assessed, 31 were diagnosed as keratosis by FFL examination, of which 11 (35.5%) were confirmed as keratosis by HPE, and the rest are shown in Figure 7.

The diagnostic accuracy of fiberoptic laryngoscopy in diagnosing keratosis is 35.5%. Among the clinically diagnosed benign lesions, the most common lesion that turned out to be malignant after histopathological examination is keratosis. Of the 159 lesions assessed, four were diagnosed as granuloma by FFL examination, of which three (75%) were histopathologically confirmed as granuloma and one (25%) as laryngitis. The diagnostic accuracy of fiberoptic laryngoscopy in diagnosing granuloma is 75%.

Of the 159 lesions examined histopathologically, 60 (37.7%) were nodules, 45 (28.3%) were polyps, 17 (10.7%) were keratosis, eight (5%) were cysts, six (3.8%) squamous cell carcinoma, five (3.1%) were granuloma, three (1.9%) were laryngitis, two (1.3%) were aspergillosis, two (1.3%) were submucous fibrosis, two (1.3%) were severe dysplasia, two (1.3%) were keratotic nodules, and seven (4.4%) were keratotic polyp. On the correlation of fiberoptic laryngoscopic diagnosis with histopathological examination, the diagnostic accuracy of FFL is 51.8% for nodules, 44.4% for polyps, 35.5% for keratosis, and 75% for granulomas. The diagnostic accuracy of FFL examination in diagnosing truly benign lesions is 94.9%. Of the six patients whose histopathological examination turned out to be a malignancy, four were clinically diagnosed as keratosis, one as papilloma, and one as congested polyp. After obtaining the results, a presumptive clinical scoring system to identify malignancy in a clinically benign-looking lesion was formulated and retrospectively applied to all the cases based on age, sex, tobacco chewing habits, smoking, and presence of congestion and edema in the vocal cords during FFL examination as shown in Table 1.

**Table 1:** Presumptive scoring system to identify malignancy in clinically benign-looking lesions FFL: flexible fiberoptic laryngoscopy

Score	0	1	2
Age (in years)	Less than 40	40-50	>50
Sex	-	Female	Male
Tobacco chewing	No	-	Yes
Smoking	No	-	Yes
Congestion and edema during FFL	Absent	-	Present

On applying the score, it was noted that seven patients had a score of more than or equal to 7. Of these, six patients were diagnosed with malignancy (squamous cell carcinoma) upon HPE. Only one lesion (0.01%) had a score of ≤7, and even that lesion was diagnosed as severe dysplasia on HPE.

## Discussion

This study provides the details of the prevalence, etiological factors, clinical presentation of patients with benign vocal cord lesions, and diagnostic potential of flexible fiberoptic laryngoscopy and its correlation with histopathology. Some of the common vocal cord lesions are vocal nodules, vocal polyps, Reinke's edema, contact ulcer, intubation granuloma, vocal cord cysts, leukoplakia, papilloma, and keratosis [2]. Vocal cord nodules may be defined as small, benign thickenings of the margins of the vocal cords, usually due to vocal abuse, and ordinarily occurring at the junction of the anterior and middle thirds of the vocal cords [5]. A vocal polyp is a benign swelling that arises from the free edge of the vocal cord. It is usually solitary but can occasionally affect both vocal cords [6]. Reinke's edema is a diffuse polypoid degeneration of the entire length of one or, more commonly, both vocal cords [7]. The potential space between the superficial layer of lamina propria and the vocal ligament forms the Reinke's space [8].

Vocal cysts are located in the lamina propria. There are two types of cysts: mucous retention cysts and epidermoid/keratin cysts [9]. They appear as opaque, spheroid masses underlying the epithelium [9], which can be unilateral or bilateral and are commonly confused with vocal nodules, especially when symmetrical. They can also be associated with other benign laryngeal lesions [10]. Laryngeal keratosis is an inflammatory disease of the epithelium of the vocal cords due to chronic hyperplasia and hyperkeratosis of the laryngeal mucosa with the resultant hyperkeratosis of the vocal cord epithelium [6]. Recurrent respiratory papillomatosis (RRP) is characterized by the development of exophytic proliferative lesions of connective tissue covered by epithelium, which affect the mucosa of the airways [11]. Although the tumors can grow anywhere in the respiratory tract, they commonly grow in the larynx, a condition called laryngeal papillomatosis [12]. Concurring with previous studies as shown in Table 2, benign vocal cord lesions were more common in male patients than in female patients. In our study, the male/female ratio is 2.3:1.

**Table 2:** Comparing gender distribution across studies

Study	Male/female ratio
Reddy et al. [13]	2.12:1
Buche et al. [14]	1.67:1
Sharma et al. [15]	2.6:1
Our study	2.3:1

A comparison of the predominant age group of our study with other studies is shown in Table 3.

**Table 3:** Comparison of age groups across studies

Studies	Age in years
Reddy et al. [13]	31-40 years
Buche et al. [14]	21-30 years
Saha et al. [16]	41-50 years
Siddapur et al. [17]	30-40 years
Our study	40-49 years

In our study, vocal nodules and polyps were seen in the older age group, and keratosis was seen in the younger age group as compared to the study done by Zhukhovitskaya et al. as shown in Table 4 [18]. The probable reason for vocal cord lesions in the younger population being less in our study may be due to increased use of technology such as mobile phones and social media with reduced habit of conversing vocally. Therefore, the prevalence of voice abuse has become lesser in the younger generation. However, the age group of patients with Reinke's edema correlates in both studies as shown in Table 4.

**Table 4:** Comparison of gender distribution between the study by Zhukhovitskaya et al. and our study

Studies	Zhukhovitskaya et al. [18]		Our study	
	Sex	Age	Sex	Age
Vocal nodules	Females	18-39 years	Females	40-49 years
Vocal polyps	Males	18-39 years	Males	40-49 years
Keratosis	Males	60 and above, followed by 40-59 years	Males	30-39 years
Reinke's edema	Females	40-59 years, followed by 60 and above	Females	40-59 years

The incidence of keratosis is predominant in the younger population as compared to the abovementioned study. This can be due to the early onset of habits such as smoking and alcohol consumption and due to increased air pollution. The predominant symptom in most patients with benign vocal cord lesions in our study is voice change (hoarseness of voice), which is the same as the previous studies [13-18]. In our study, most of the patients were business people (33.6%) by profession, while in two of the other studies [13-17], patients were predominantly housewives. However, in our study, the second most common profession was teachers (24%), and the third was housewives (16.8%). The most common risk factor for developing vocal cord lesions in our study was voice abuse (57.6%), which correlates with all the previous studies on benign vocal cord lesions [13-18]. The most common lesion clinically diagnosed in our study is vocal cord polyp (61%), followed by vocal cord nodules (14%) as against other studies where vocal cord nodules are the most common lesion [13-18]. This discrepancy between our study and other studies may be because our center is a tertiary care center where patients mostly come for second opinions. A huge volume of patients with early vocal nodules who are treated with voice therapy alone are missed as they do not require a second opinion.

Regarding the severity of symptoms of different benign vocal cord lesions, the mean VHI-10 scores of nodules and polyps were calculated and compared. The mean VHI-10 scores of vocal nodules were slightly on the higher side compared to the mean VHI-10 scores of vocal polyps. A similar study done by Huang et al. stated that there was no significant difference between the VHI-10 scores of the two abovementioned lesions [19]. In our study, the diagnostic accuracy of fiberoptic laryngoscopy in diagnosing a truly benign lesion as benign is 94.9% as compared to 91.5% in the study conducted by Nerurkar and Garg, which was done to correlate rigid Hopkins rod telescope diagnosis with histopathological diagnosis [3]. It is noted that among the six cases that were diagnosed as malignant after histopathological examination, four were diagnosed as keratosis by FFL. A clinician should have a high index of suspicion for keratotic lesions. Newer technologies such as narrow band imaging or optical coherence tomography can be used in such cases to identify these malignant lesions that clinically look benign.

On comparing the reliability of preoperative diagnosis in our study with that of the study done by Nerurkar and Garg, it was noted that the reliability of preoperative diagnosis for vocal cord polyps is higher in our study, while it is lesser compared to the abovementioned study in all other lesions. This discrepancy can be attributed to less number of cases in the abovementioned study as compared to our study and also due to observer-based variations [3]. In a study done to observe inter-observer and intra-observer variations in clinical diagnosis of benign vocal cord lesions, images of the larynx with benign vocal cord lesions were shown to many ENT specialists, and their diagnoses were compared. It was noted that strikingly, not in one patient was there total agreement on clinical diagnoses. A consensus of at least 75% occurred in only five out of 45 pairs of slides: once for Reinke's edema, twice for polyps, and twice for vocal cord nodules. The greatest consensus was achieved in patients with vocal cord nodules: 88% in two patients. When the same image was shown to the same observer after a long time span, the mean intra-observer kappa was calculated to be 0.59 [20].

This discrepancy can also be due to observer bias among the pathologists. Histopathological diagnoses are not always concordant with clinical diagnoses. This disagreement may be justified by the fact that these pathologies are theoretically different. While these lesions are well differentiated for otolaryngologists, pathologists do not see this differentiation. Microscopically, nodules and polyps are defined as identical lesions resulting from phonotrauma with or without stress, inflammatory irritation, and allergic factors that develop mainly in the anterior third of the vocal folds causing hoarseness. The microscopic appearance depends on the stage of the lesion (whether it is called a polyp or a nodule); in the beginning, there is edema, fibroblast proliferation, and, later, vascular and stromal hyalinization [21].

Cipriani et al., in their study, stated that despite the apparent distinctiveness of the clinical nomenclature of benign vocal cord lesions, low inter- and intra-observer diagnostic agreement has been reported. A total of 78 non-neoplastic lesions of the vocal cord were reviewed by two pathologists. In 46 cases with prebiopsy stroboscopic images, two otolaryngologists classified each lesion as a polyp, nodule, Reinke's edema, cyst, or other. They agreed in 43% (n = 20, 13 polyps, 5 nodules, 1 Reinke's edema, and 1 other) and disagreed in 57% (n = 26). There was no histologic feature that reliably distinguished among the lesions. Cysts were distinctive, as all were epithelial-lined. The clinicopathologic classification of benign laryngeal lesions is neither clinically reproducible nor histologically unique [22].

As our hospital is a tertiary care center, many patients with vocal nodules may present late. Due to the chronicity of the lesion, long-standing trauma to the nodule, and superadded inflammation, they may clinically appear as a polyp during fiberoptic laryngoscopy. A presumptive scoring system to identify the probability of malignancy preoperatively was done retrospectively in our study. It showed a reliability of 86%. Such clinical scoring will help physicians identify patients with a higher risk of malignancy. Such patients with high scores should be advised to undergo a detailed microscopic examination under anesthesia and biopsy if necessary.

Limitations

This study is conducted in a tertiary care center, where patients mostly come for second opinions after being advised surgery elsewhere. Hence, a significant number of early vocal cord lesions could have been missed. The presumptive scoring system to identify malignancy in clinically benign-looking lesions mentioned in this study is formed retrospectively after obtaining the histopathology report. The number of patients who had a malignant pathology was much less, six against 112 benign pathology. This scoring system needs validation by testing the same prospectively on a larger number of patients.

## Conclusions

Benign vocal cord lesions are one of the most common cases seen in ENT practice. Vocal polyp followed by vocal nodules was found to be the most common etiology with significant voice handicaps. Although histopathological examination can confirm a definite diagnosis, our study shows promising results with the flexible fiberoptic laryngoscopy. Therefore, the study recommends the use of flexible fiberoptic laryngoscopy as a screening tool for the diagnosis of vocal disorders in order to decide on the mode of management at the earlier stage of the disease and also to prevent the development of complications.
